# Anlotinib Inhibits Tumor Angiogenesis and Promotes the Anticancer Effect of Radiotherapy on Esophageal Cancer through Inhibiting EphA2

**DOI:** 10.1155/2022/5632744

**Published:** 2022-08-31

**Authors:** Zhenlin Gu, Weiguo Zhu, Wanwei Wang, Yingying Xu, Lei Jiang, Jiasheng Huang, Jing Huang

**Affiliations:** ^1^Department of Vascular Surgery, The Affiliated Huaian No. 1 People's Hospital of Nanjing Medical University, Huaian 223300, Jiangsu, China; ^2^Department of Radiation Oncology, The Affiliated Huaian No. 1 People's Hospital of Nanjing Medical University, Huaian 223300, Jiangsu, China; ^3^Department of Interventional Radiology, The Affiliated Huaian No. 1 People's Hospital of Nanjing Medical University, Huaian 223300, Jiangsu, China

## Abstract

**Background:**

Anlotinib is a novel multitarget tyrosine kinase inhibitor for tumor angiogenesis and has antitumor activity in a variety of solid tumors. Given that, our study was designed to unearth the mechanism of anlotinib in radioresistant esophageal cancer (EC) cells.

**Methods:**

Radioresistant EC cell lines TE-1R and KYSE-150R were established by multiple fractionated irradiation. Detection of cell proliferation was governed by the MTT assay, angiogenesis by the tube formation assay, and cell migration and invasion by the transwell assay. Lastly, RT-qPCR Western blotting was employed to detect the expression of related genes. Cancerous cells showing tumor growth were then detected by tumor xenografts in mice.

**Results:**

Radioresistant EC cell lines TE-1R and KYSE-150R were successfully established. Anlotinib downregulated EphA2 inhibited proliferation, angiogenesis, migration, and invasion of radioresistant EC cells *in vitro*. The up-regulated expression of EphA2 in both EC cell lines and radioresistant EC cells, along with anlotinib, in turn, inhibited the expression of EphA2 in radioresistant EC cells. Inhibiting EphA2 also enhanced anlotinib-mediated effects on radioresistant EC cells, so as to restrain cell proliferation, angiogenesis, migration, and invasion. Correspondingly, overexpression of EphA2 is capable of reversing the therapeutic effect of anlotinib on radioresistant EC cells. Also, anlotinib enhances the inhibitory effect of irradiation on mice.

**Conclusion:**

It is concluded that anlotinib inhibits EphA2 expression, thereby suppressing angiogenesis and resensitizing EC cells to radiotherapy, providing another perspective to overcome radioresistance in EC.

## 1. Introduction

As a heterogeneous malignancy, esophageal cancer (EC) is mostly diagnosed in advanced stages and esophageal squamous cell carcinoma (ESCC) and accounts for most cases of the disease [1]. Smoking, alcohol consumption, gastroesophageal reflux disease, obesity, and diet are common risk factors for EC [2]. EC is usually asymptomatic in the early stages, and in advanced disease one may complain of heartburn unresponsive to medication, unconscious weight loss, progressive dysphagia, signs of blood loss, chest pain, and odynophagia [3]. Multimodality approaches such as endoscopic mucosal resection and endoscopic submucosal dissection, surgical treatment, neoadjuvant and adjuvant chemotherapy, as well as concurrent chemoradiotherapy have been developed for the treatment of EC [4]. However, tumor-associated microenvironmental factors and cellular mechanisms may somehow lead to radioresistance [5]. Thus, dealing with radioresistance may be a practical approach to manage EC. Blood vessel normalization in tumors could reduce tumor uptake. Intratumoral accumulation, [6] tumor blood vessel normalization, and ES radiotherapy need further research studies.

Anlotinib is an orally administered tyrosine kinase inhibitor that is designed to inhibit angiogenesis and growth of tumors [7]. Anlotinib could reduce blood vessel sprout and microvessel density (MVD), and restrain migration and tube formation in tumors [8]. In fact, anlotinib has great therapeutic efficacy in treating cancers, such as advanced nonsmall cell lung cancer (NSCLC), advanced soft tissue sarcoma, and metastatic renal cell carcinoma [9]. In ESCC, it has been reported that anlotinib combined with radiotherapy and chemotherapy has strong antitumor effects on patient-derivedxenografts-bearing mice [10]. In a clinical trial, it has been found that anlotinib combined with chemotherapy could improve the survival of patients with advanced ESCC [11]. Considering the essence of anlotinib, tyrosine kinase receptors attracted our attention to determine the mechanism of anlotinib in EC. Belonging to the tyrosine kinase receptor group, EphA2 is abundantly produced in tumors and the regulation of EphA2 confers a potential in managing tumors [12]. EphA2 is a tumor-associated surface antigen of chimeric antigen receptor used in the treatment of ESCC [13]. It has been further analyzed that regulating EphA2 expression mediates vasculogenic mimicry of EC cells [14]. In ESCC samples after radiotherapy, the genomic profile of EphA2 is altered and the absence of mutation of EphA2 confers radioresistance [15]. In endometrial cancer, EphA2 overexpression is positively correlated with high VEGF expression, which is associated with angiogenesis and disease-specific survival of patients [16]. Referring to these reports, we assumed that anlotinib suppresses radioresistance and tumor angiogenesis of EC cells through inhibiting EphA2, and it may renew the mechanism underlying radioresistance in EC and provide for therapeutic reference.

## 2. Materials and Methods

### 2.1. Ethics Statement

Animal experiments were reviewed and approved by the animal ethics committee of “The Affiliated Huaian No.1 People's Hospital of Nanjing Medical University.”

### 2.2. Cell Culture

Human normal esophageal epithelial cells (THEECs) and EC cell lines TE-1 and KYSE-150 (ATCC, VA, USA) were kept in Roswell Park Memorial Institute (RPMI)-1640 (10% fetal bovine serum [FBS], 100 unit/mL penicillin, and 100 mg/mL streptomycin). The media were all provided by Gibco (NY, USA).

### 2.3. Induction of Radioresistance in EC Cells

Radioresistant EC cell lines (TE-1R and KYSE-150R) were induced through multiple fractionated irradiation [17]. TE-1 and KYSE-150 cells (1.5 × 10^6^ cells) in a culture flask (25 cm^2^) were irradiated with 1 Gy X-ray, immediately supplemented with a fresh medium, and were grown to 90% confluence. Then, cells were cultured in a new culture flask to 50% confluence and treated with a second irradiation. Totally, cells were irradiated at 1 Gy three times, 2 Gy three times, and 4 Gy three times.

### 2.4. Colony Formation Assay

A colony formation assay was utilized to assess the radioresistance of parental and resistant EC cells. Parental and resistant EC cells in the log phase were trypsinized and seeded into 100-mm petri dishes. Upon cell adherence, cells were irradiated with 0, 2, 4, 6, 8, and 10 Gy X-ray, respectively, and were continuously cultured for 12 days to form cell colonies.

### 2.5. Cell Transfection

Cells in the log phase were cultured on a 6-well plate containing RPMI-1640 (2 × 10^5^ cells/well). Cells at 90% confluence were transfected with EphA2-negative control (CTRL), siRNA-EphA2, or overexpression (OE)-EphA2 (GenePharma, Shanghai, China) via Lipofection™ (InivoGene, CA, USA). Three replicate wells were set.

### 2.6. Anlotinib Treatment

Cells were treated with anlotinib (CTTQ, Jiangsu, China) at 2, 4, and 8 *μ*mol/L, respectively, for 48 h. A control was established with cells treated with normal saline [18].

### 2.7. 3-(4, 5-Dimethylthiazol-2-yl)-2,5-diphenyltetrazolium Bromide (MTT) Assay

Cells were placed in a 96-well plate at 3 × 10^3^ cells/well. After 48 h, cells were combined with MTT solution at 20 *μ*L/well (Beyotime, Shanghai, China) for 4 h, and treated with dimethyl sulfoxide at 100 *μ*L/well. The D value at 490 nm was recorded on an automatic microplate reader (Tecan M200, TECAN, Switzerland).

### 2.8. Tube Formation Assay

Cells were cultured in a serum-free medium for 24 h and then in a medium containing 10% FBS. Then, the supernatant was centrifuged at 1000 r/min and filtered through a filter (0.22 *μ*m) to obtain the conditioned medium (CM), which was preserved at 4 °C. A mixture (40 *μ*L) made by the CM and Matrigel (1 : 1) was spread on a 96-well plate overnight and incubated with human umbilical vein endothelial cell suspension (1 × 10^5^ cells/mL) at 200 *μ*L/well. The formed tubes were observed and counted in 4 fields of view under a microscope (Olympus, Tokyo, Japan) [18].

### 2.9. Transwell Assay

Cells were prepared into a single cell suspension with serum-free Dulbecco's Modified Eagle Medium (DMEM). The cell suspension (100 *μ*L, 3 × 10^5^ cells/mL) was added to the upper side of the Transwell chamber (Corning, N.Y., USA). Matrigel (BD Company, NJ, USA) was used for the invasion assay but not for the migration assay. The bottom chamber was supplemented with 10% FBS-DMEM (600 *μ*L). After 24 h, cells were fixed with 95% ethanol, stained with crystal violet, and counted under a microscope.

### 2.10. Tumor Xenografts in Nude Mice

Male and female BALB/c(nu/nu) nude mice (4–6 weeks old; 15–18 g) were provided by Beijing Vital River Laboratory Animal Technology Co., Ltd. (Beijing, China). Mice were housed in specific pathogen-free-level animal barriers (18–23°C, humidity 50–60%, 12 h day/night alternate, disinfected food and water). A week later, the skin on the left back of the mice was sterilized with ethanol, and the mice were subcutaneously injected with 100 *μ*L of cell suspension (1 × 10^6^ cells/mL) into the back. In the following 2 weeks, the general condition of the mice and the local condition of the injection site were observed. The mice were divided into three groups: KYSE-150R group, KYSE-150R + X-ray group, and KYSE-150R + Anlotinib + X-ray group. The mice in the KYSE-150R + Anlotinib + X-ray group were given anlotinib at 1.5 mg/kg by intragastric administration for 2 weeks. The mice in the KYSE-150R + X-ray group and KYSE-150R + Anlotinib + X-ray group were irradiated with 6 Gy X-rays every week. At 4 weeks postinjection, the mice were euthanized, the excised tumors were weighed, and tumor volume was measured [18].

### 2.11. Reverse Transcription Quantitative Polymerase Chain Reaction (RT-qPCR)

After extraction of total RNA in tissues and cells by Trizol (Invitrogen, CA, USA), RNA concentration was determined with Nanodrop 2000 (Thermo Fisher Scientific, MA, USA). RNA was reverse-transcribed to cDNA using the PrimeScript RT kit (Takara, Kyoto, Japan). Using the SYBR Premix Ex Taq kit (Tli RNase H Plus) kit (Takara), real-time PCR was performed on an ABI7500 (Thermo Fisher Scientific). EphA2 expression was calculated by the 2^-△△Ct^ method [16] and normalized to glyceraldehyde-3-phosphate dehydrogenase (GAPDH). The primers (GenePharma) are shown in Table 1.

### 2.12. Western Blot Assay

After extraction of protein from tissues and cells, protein concentration was measured by the bicinchoninic acid method. The protein was mixed with loading buffer at 2 : 1 and denatured. After separation by sodium dodecyl sulfate-polyacrylamide gel electrophoresis, the protein was transferred to a polyvinylidene fluoride membrane and combined with primary antibodies EphA2 (1 : 2000, Thermo Fisher Scientific), VEGF (1 : 2000, Abcam), basic fibroblast growth factor (bFGF; 1 : 1000, Abcam), and GAPDH (1 : 1000, Millipore, MA, USA). Afterward, an HRP-labeled secondary antibody (1 : 5,000, Abcam) reacted with the membrane which was then developed by enhanced chemiluminescence. GAPDH was referred to as an internal control. Target protein expression was calculated by the gray analysis software.

### 2.13. Statistical Analysis

Data were assessed with SPSS 21.0 (IBM, NY, USA) and measurement data were expressed as mean ± standard deviation. Measurement data in normal distribution were compared by the *t*-test between the two groups. One-way analysis of variance (ANOVA), followed by Tukey's multiple comparisons test was applied to analyze data among multiple groups. At *P* < 0.05, statistical significance was established.

## 3. Results

### 3.1. Induction of Radioresistance in EC Cells

**Figure 1:**
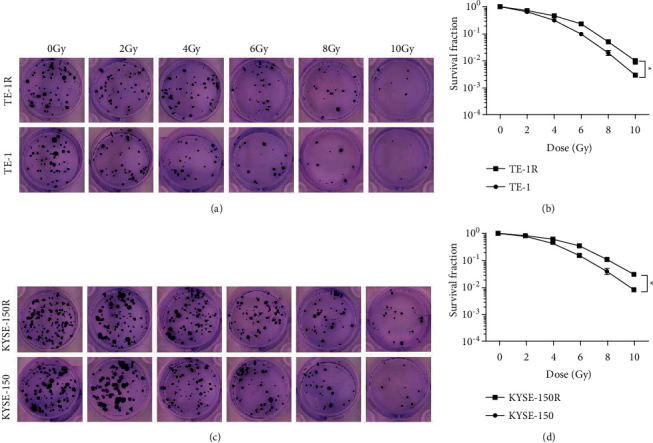
Induction of radioresistance in EC cells. (a) Colony-forming ability of TE-1 and TE-1R cells irradiated with different doses, (b) survival curve of TE-1 and TE-1R cells irradiated with different doses, (c) colony-forming ability of KYSE-150 and KYSE-150R cells irradiated with different doses, and (d) survival curve of KYSE-150 and KYSE-150R cells irradiated with different doses.  ^*∗*^*P* < 0.05 and  ^*∗∗*^*P* < 0.01; repetition = 3; the data were expressed in the form of mean ± standard deviation and were compared by the *t*-test.

Colony formation assay was applied to assess the radioresistance of parental and radioresistant EC cells. The outcomes indicated that after irradiation at 0, 2, 4, 6, 8, and 10 Gy, respectively, for 12 days, the number of formed colonies decreased with the increase in the irradiation dose. Also, when irradiated at the same dose, the number of colonies of radioresistant EC cells increased as compared to parental EC cells. Since the results indicated that radioresistant EC cells had stronger radioresistance and colony-forming ability, it was confirmed that radioresistant EC cell lines TE-1R and KYSE-150R were successfully established (Figures 1(a)–1(d)).

### 3.2. Anlotinib Inhibits Proliferation, Angiogenesis, Migration, and Invasion of Radioresistant EC Cells

**Figure 2:**
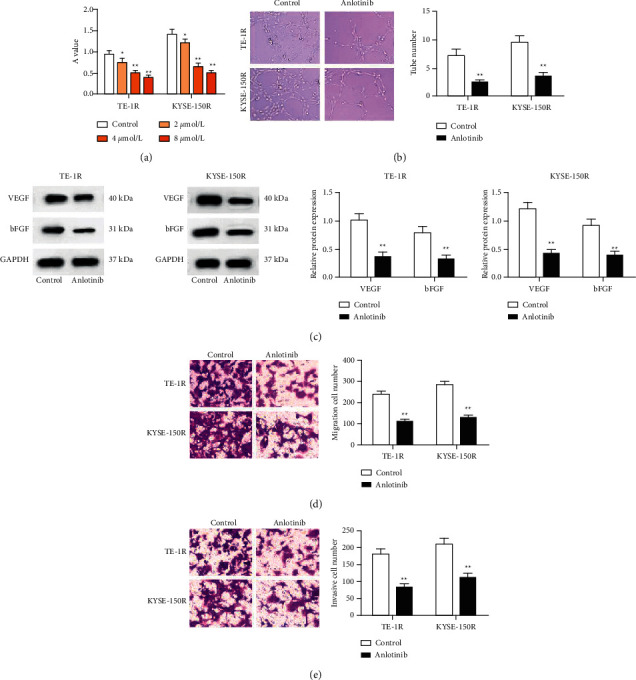
Anlotinib inhibits proliferation, angiogenesis, migration, and invasion of radioresistant EC cells. (a) The MTT assay detected cell proliferation, (b) the tube formation assay detected cell angiogenesis, (c) Western blot detected VEGF and bFGF expression in cells, (d) the transwell assay detected cell migration, and (e) the transwell assay detected cell invasion.  ^*∗*^*P* < 0.05 and  ^*∗∗*^*P* < 0.01; repetition ^*∗*^,  ^*∗∗*^ = 3; the data were expressed in the form of mean ± standard deviation. Data were compared by the *t*-test (two groups), or one-way ANOVA (multiple groups) and Tukey's multiple comparisons test (pairwise comparison).

Anlotinib has antitumor activity in various solid tumors, however, its effect on the anticancer effect of radiotherapy in EC was unclear at times. To further explore this issue, we established TE-1R and KYSE-150R cell lines and treated the cells with different concentrations of anlotinib (2, 4, and 8 *μ*mol/L). It was found from the MTT assay that after anlotinib treatment, the proliferation of TE-1R and KYSE-150R cells was impaired in a concentration-dependent manner (Figure 2(a)). The inhibitory effect of anlotinib on proliferation was more effective at 4 *μ*mol/L; therefore, anlotinib at 4 *μ*mol/L was used for later experiments.

In tube formation and transwell assays, along with the Western blot assay, we disclosed that after anlotinib treatment, tumor angiogenesis, migration, and invasion of TE-1R and KYSE-150R cells were inhibited, and protein expression of angiogenesis-related factors VEGF and bFGF was reduced (Figures 2(b)–2(e)).

### 3.3. EphA2 Expression Is Raised in Radioresistant EC Cells

**Figure 3:**
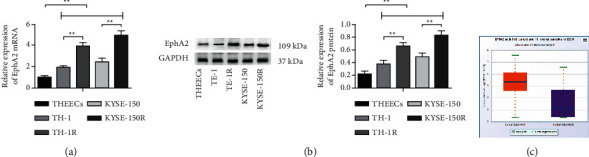
EphA2 expression is raised in radioresistant EC cells. (a) RT-qPCR detected EphA2 expression in THEECs, EC cell lines (TE-1 and KYSE-150), and radioresistant EC cells (TE-1R and KYSE-150R), (b) Western blot detected EphA2 expression in THEECs, EC cell lines (TE-1 and KYSE-150), and radioresistant EC cells (TE-1R and KYSE-150R), and (c) Starbase predicted that EphA2 was upregulated in EC.  ^*∗*^*P* < 0.05 and  ^*∗∗*^*P* < 0.01; repetition ^*∗*^,  ^*∗∗*^ = 3; the data were expressed in the form of mean ± standard deviation and compared by one-way ANOVA and Tukey's multiple comparisons tests.

EphA2, a tyrosine kinase receptor, has been reported to be upregulated in ESCC [13]. In the present study, we applied RT-qPCR and Western blot to measure EphA2 expression in cells. The outcome reflected that EphA2 expression was higher in TE-1 and KYSE-150 cells than in THEECs, and was higher in TE-1R and KYSE-150R cells than in TE-1 and KYSE-150 cells (Figures 3(a) and 3(b)). In addition, we also found that EphA2 was upregulated in EC on the Starbase website (Figure 3(c)).

### 3.4. Inhibiting EphA2 Enhances Anlotinib-Mediated Effects on Radioresistant EC Cells

**Figure 4:**
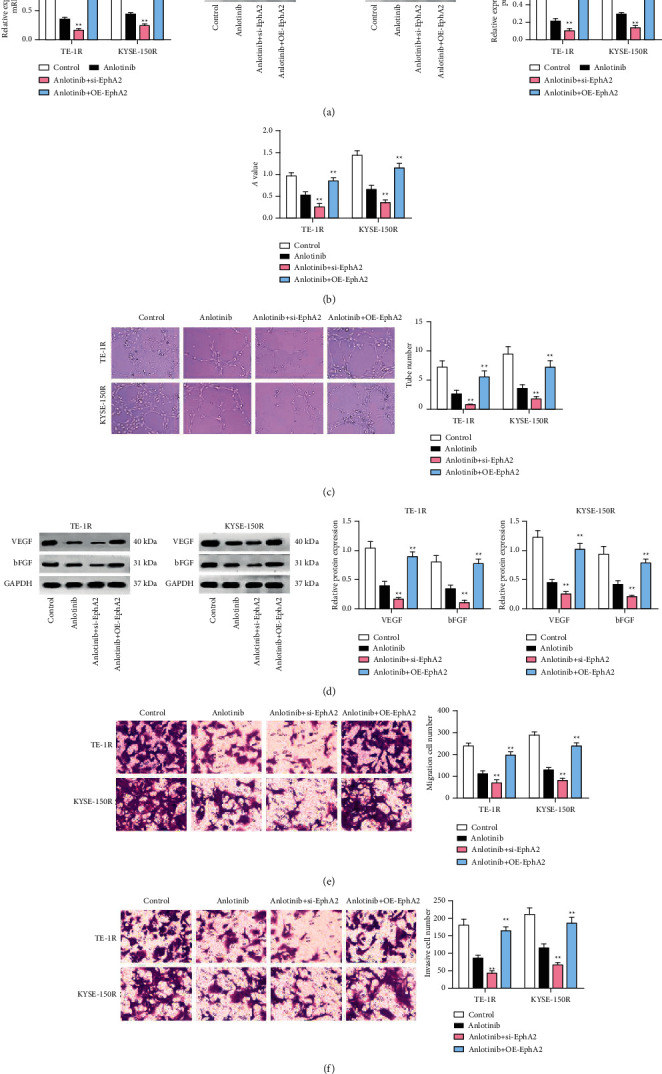
Inhibiting EphA2 enhances anlotinib-mediated effects on radioresistant EC cells. (a) RT-qPCR and Western blot detected EphA2 expression in cells, (b) the MTT assay detected cell proliferation, (c) the tube formation assay detected cell angiogenesis, (d) Western blot detected VEGF and bFGF expression in cells, (e) the transwell assay detected cell migration, and (f) the transwell assay detected cell invasion.  ^*∗*^*P* < 0.05 and  ^*∗∗*^*P* < 0.01; repetition ^*∗*^,  ^*∗∗*^ = 3; the data were expressed in the form of mean ± standard deviation and compared by one-way ANOVA and Tukey's multiple comparisons tests.

We utilized RT-qPCR and Western blot to test EphA2 expression in TE-1R and KYSE-150R cells and revealed that anlotinib treatment reduced EphA2 expression (Figure 4(a)). Then, we applied siRNA-EphA2 or OE-EphA2 to downregulate or upregulate EphA2 expression in TE-1R and KYSE-150R cells, and we treated these cells with anlotinib at 4 *μ*mol/L. Subsequently, the experimental data from *in vitro* cell function experiments indicated that OE-EphA2-mediated the upregulation of EphA2 and reversed the inhibitory effect of anlotinib on VEGF and bFGF protein expression, as well as on proliferation, tumor angiogenesis, and migration, and on invasion abilities of TE-1R and KYSE-150R cells. By contrast, siRNA-EphA2-induced downregulation of EphA2 which further enhanced anlotinib-mediated effects on TE-1R and KYSE-150R cells (Figures 4(b)–4(f)).

### 3.5. Downregulating EphA2 Depresses Proliferation, Angiogenesis, Migration, and Invasion of Radioresistant EC Cells

**Figure 5:**
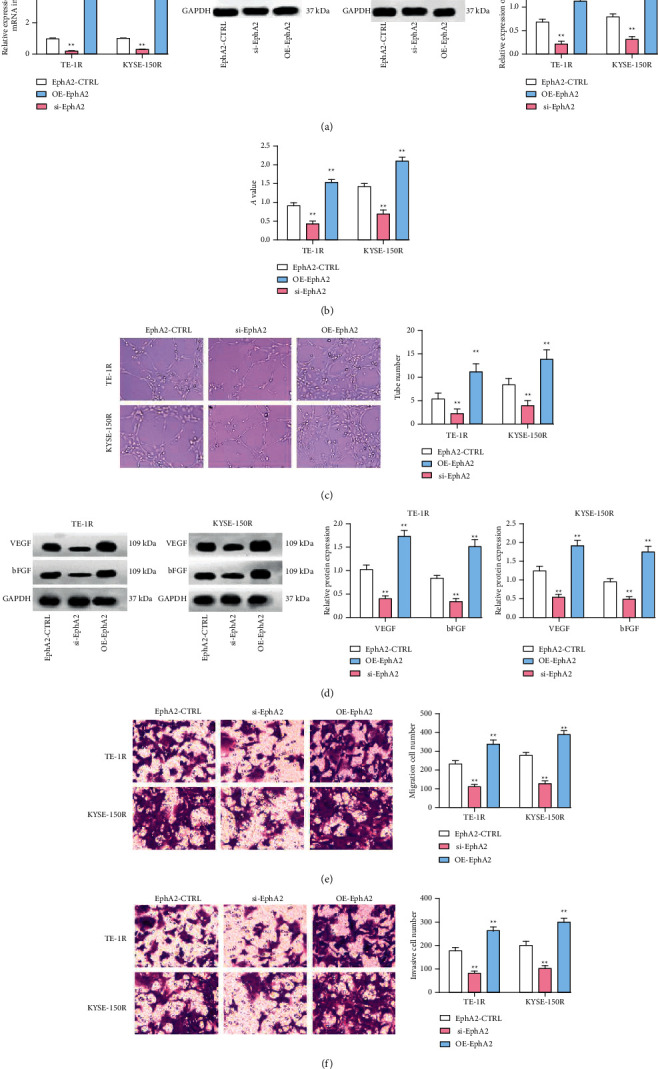
Downregulating EphA2 depresses proliferation, angiogenesis, migration, and invasion of radioresistant EC cells. (a) RT-qPCR and Western blot detected EphA2 expression in cells, (b) the MTT assay detected cell proliferation, (c) the tube formation assay detected cell angiogenesis, (d) Western blot detected VEGF and bFGF expression in cells, (e) the transwell assay detected cell migration, and (f) the transwell assay detected cell invasion.  ^*∗*^*P* < 0.05 and  ^*∗∗*^*P* < 0.01;  ^*∗*^,  ^*∗∗*^repetition = 3; the data were expressed in the form of mean ± standard deviation and compared by one-way ANOVA and Tukey's multiple comparisons tests.

Next, we further explored the effect of EphA2 on cells and transfected siRNA-EphA2 or OE-EphA2 into TE-1R and KYSE-150R cells. At first, RT-qPCR and Western blot were employed to verify that EphA2 expression in cells was successfully downregulated or upregulated by siRNA-EphA2 or OE-EphA2 (Figure 5(a)). Next, through *in vitro* cell function experiments, we noticed that silencing EphA2 reduced proliferation, tumor angiogenesis, migration, and invasion, as well as VEGF and bFGF protein expression in cells, while restoring EphA2 had opposite effects (Figure 5(b)–5(f)).

### 3.6. Anlotinib Suppresses Growth of Radioresistant EC Cells *In Vivo*

**Figure 6:**
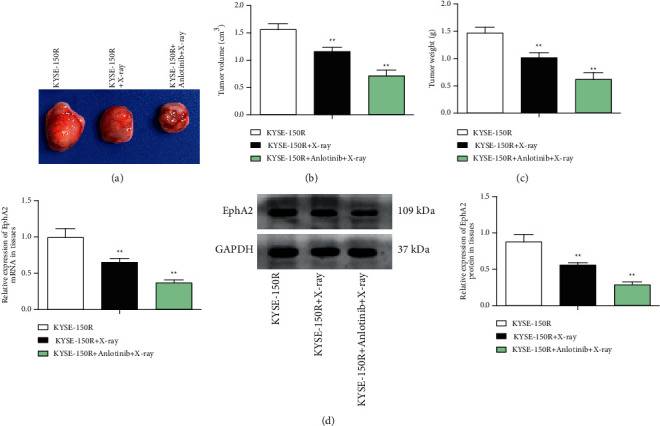
Anlotinib suppresses the growth of radioresistant EC cells *in vivo*. (a) Representative images of tumors, (b) tumor volume in nude mice, (c) tumor weight in nude mice, and (d) RT-qPCR and Western blot detected EphA2 expression in tumors of nude mice.  ^*∗*^*P* < 0.05 and  ^*∗∗*^*P* < 0.01;  ^*∗*^,  ^*∗∗*^*n* = 5; the data were expressed in the form of mean ± standard deviation and compared by one-way ANOVA and Tukey's multiple comparisons tests.

The tumor formation rate of 24 nude mice was 100%, and no natural death occurred during the experiment. For mice exposed to irradiation, it was recognized that tumor volume, weight, and EphA2 expression were all suppressed. Then, further treatment with anlotinib was found to enhance the inhibitory effects of irradiation on mice (Figures 6(a)–6(d)).

## 4. Discussion

Radiation has an established role in definitive, palliative, and neoadjuvant environments, having a vital effect on the treatment of local EC [19]. Multiple drugs have been introduced to overcome radioresistance in EC, including anlotinib. In our research, we have recognized the therapeutic efficacy of anlotinib and further disclosed the underlying mechanism of anlotinib by regulating EphA2 in EC. Collectively, anlotinib inhibited tumor angiogenesis of radioresistant EC cells by inhibiting EphA2.

To specify the action of anlotinib in radioresistance of EC, we administrated anlotinib at 4 *μ*mol/L to treat radioresistant EC cells and observed its inhibitory impacts on cellular proliferation, angiogenesis, migration, and invasion, as well as tumor growth. In a case report, it has been observed that administration of anlotinib has a better response for the fourth-line therapy and prolongs the overall survival time of patients with ESCC [11]. In another clinical trial, it has been noticed that combined administration of nivolumab and anlotinib as a second-line therapy could improve the physical condition of the patient with advanced ESCC [20]. In addition to that, a recent report has highlighted that anlotinib and chemoradiotherapy in combination have the ideal antitumor effect to suppress the process of ESCC in mice [10]. Besides, a double-blind randomized phase 2 trial has mentioned that the use of anlotinib has a great advantage in improving progression-free survival (PFS) of patients within recurrent and metastatic ESCC [21]. A case report has observed that for ESCC patients with failed immunotherapy course, their survival is greater than 19 months, and the overall patient survival is greater than 32 months after a fourth-line therapy (anlotinib combined with chemotherapy) [11]. Anlotinib combined with concurrent chemoradiotherapy improves the clinical efficacy and safety of locally advanced ESCC patients [22]. Not only limited to EC but treatment with anlotinib works actively in other cancer types. For instance, treatment with anlotinib in thyroid cancer cells causes impairments in cell viability and migration *in vitro* and tumor growth *in vivo* [23]. Moreover, some studies have emerged on the regulatory mechanism of anlotinib in suppressing tumorigenesis. It is revealed that anlotinib could limit lung cancer cells to proliferate, invade, and migrate and can limit tumor growth by blocking the mitogen-activated protein kinase/extracellular signal-regulated kinase (ERK) pathway [24]. Other than that, anlotinib-induced inhibition of proliferation, migration, invasion, and tube formation, as well as tumorigenicity *in vivo* is recognized in colorectal cancer through suppressing the AKT/ERK pathway [18]. Our study also mentioned that anlotinib also exhibited great effects on promoting the efficacy of radiotherapy in EC. Consistently, it is noted that in the setting of lung cancer, the synergism of radiotherapy and anlotinib is more effective to suppress cell proliferation and tumor cell growth than the administration of anlotinib alone [25]. Overall, anlotinib is a promising drug for managing the process of cancer and improving the survival of cancer patients; moreover, anlotinib and radiotherapy synergistically function to control the tumorigenic activities of malignant cells.

Next, we studied that EphA2 was upregulated in EC cell lines and radioresistant EC cells, and further validated that anlotinib suppressed EphA2 expression in radioresistant EC cells. Subsequently, we performed cell function assays and finally uncovered that upregulating EphA2 enhanced the proliferation, invasion, migration, and angiogenesis of radioresistant EC cells. On the contrary, downregulating EphA2 had opposite effects. Deeply, we analyzed the synergism of EphA2 and anlotinib and revealed that inhibiting EphA2 strengthened the effects of anlotinib on radioresistant EC cells. In fact, phosphotyrosine profiling has indicated that EphA2 expression is raised in ESCC, and knocking down EphA2 could decrease the proliferation and invasion of malignant cells [26]. Other researchers have also identified the role of EphA2 in various tumors. For example, EphA2 expression is elevated in small-cell lung cancer, and suppression of EphA2 has the ability to restrain cell proliferation [27]. Concerning the regulatory role of EphA2 in cancer radioresistance, it has been described that blocking EphA2 could suppress the radioresistance of NSCLC cells, as well as the migration, proliferation, and invasion of malignant cells [28]. It is known that miR-200c-induced radiosensitivity, as well as invasion, migration, and tube formation reduction, is associated with EphA2 downregulation in human cancer cells [29]. VEGF and bFGF are both proangiogenic factors [30]. Regarding the molecular mechanism of VEGF and bFGF inhibition by EphA2, there are studies explaining that EphA2 is involved in the p38 MAPK/VEGF pathway [31, 32] and EphA2 promotes bFGF expression by activating the AKT signaling pathway [33], suggesting that EphA2 may positively regulate the expression of VEGF and bFGF through the p38 MAPK and AKT pathways.

## 5. Conclusion

The research concludes in a manner that it provides a novel perspective on the regulatory mechanism of anlotinib in EC, and delineates that anlotinib could circumstantially inhibit EphA2 expression, thus suppressing angiogenesis and resensitizing EC cells to radiotherapy. However, our study is at the preclinical level, and many efforts are required to develop the results in clinics. [34].

## Data Availability

The data used to support the findings of this study are included within this article.
